# Malaria Trigram: improving the visualization of recurrence data for malaria elimination

**DOI:** 10.1186/s12936-021-03964-z

**Published:** 2021-10-30

**Authors:** Cleber Matos de Morais, Kayo Henrique de Carvalho Monteiro, Jose Diego Brito-Sousa, Wuelton Marcelo Monteiro, Vanderson Souza Sampaio, Patricia Takako Endo, Judith Kelner

**Affiliations:** 1 Centro de Informática, Universidade Federal de Pernambuco, Recife, Brazil; 2 Departamento de Mídias Digitais, Universidade Federal da Paraíba, João Pessoa, Brazil; 3 Programa de Pós-Graduação em Engenharia da Computação, Universidade de Pernambuco, Recife, Brazil; 4 Universidade do Estado do Amazonas, Manaus, Brazil; 5 Fundação de Medicina Tropical Dr. Heitor Vieira Dourado, Manaus, Brazil

**Keywords:** Malaria, Elimination, Surveillance, Recurrence, Visualization

## Abstract

**Background:**

Although considerable success in reducing the incidence of malaria has been achieved in Brazil in recent years, an increase in the proportion of cases caused by the harder-to-eliminate *Plasmodium vivax* parasite can be noted. Recurrences in *P. vivax* malaria cases are due to new mosquito-bite infections, drug resistance or especially from relapses arising from hypnozoites. As such, new innovative surveillance strategies are needed. The aim of this study was to develop an infographic visualization tool to improve individual-level malaria surveillance focused on malaria elimination in the Brazilian Amazon.

**Methods:**

Action Research methodology was employed to deal with the complex malaria surveillance problem in the Amazon region. Iterative cycles were used, totalling four cycles with a formal validation of an operational version of the Malaria Trigram tool at the end of the process. Further probabilistic data linkage was carried out so that information on the same patients could be linked, allowing for follow-up analysis since the official system was not planned in such way that includes this purpose.

**Results:**

An infographic user interface was developed for the Malaria Trigram that incorporates all the visual and descriptive power of the Trigram concept. It is a multidimensional and interactive historical representation of malaria cases per patient over time and provides visual input to decision-makers on recurrences of malaria.

**Conclusions:**

The Malaria Trigram is aimed to help public health professionals and policy makers to recognise and analyse different types of patterns in malaria events, including recurrences and reinfections, based on the current Brazilian health surveillance system, the SIVEP-Malária system, with no additional primary data collection or change in the current process. By using the Malaria Trigram, it is possible to plan and coordinate interventions for malaria elimination that are integrated with other parallel actions in the Brazilian Amazon region, such as vector control management, effective drug and vaccine deployment strategies.

## Background

The malaria burden has been substantially reduced over recent years. Improvements in diagnosis, therapeutic and vector control strategies have led to a remarkable decrease in both infection and deaths worldwide [1]. In Brazil, most of malaria cases are restricted to the Amazon region (more than 99%) and although the transmission has decreased over the past years, it remains high and poses an additional challenge to the goal of elimination [2, 3].

Besides the high transmission, and although *Plasmodium falciparum* infections still contribute to a small fraction of cases, *Plasmodium vivax* infections prevails in the entire region, with almost 90% of total cases [4, 5]. This constitutes a pitfall for malaria elimination since recurrences, common for this species, are prevalent in the region [6]. However, malaria transmission is not homogeneous within the Brazilian Amazon, which is influenced by factors including deforestation, climate, economics, demographics, and the availability of health services [5, 7, 8].

Because of the aforementioned regional differences in malaria epidemiology in Brazil, the Brazilian Ministry of Health’s (MoH) NMCP (National Malaria Control Program) has two information systems for reporting malaria cases: the SINAN (Notification Information System) for the extra-Amazon region, with a very low transmission level; and the SIVEP-Malária (Malaria Epidemiological Surveillance Information System), for the Brazilian Amazon region, which is one of the most capable malaria surveillance information systems in the world [6]. The system was developed in 2002 and released in 2003 and has been enhanced ever since. However, it was planned and developed for a high transmission scenario for which the burden of disease is of more interest than the patient follow-up. Nevertheless, as transmission decreases, strategies focused on individual data rather than community level data are needed [9].

The Global Technical Strategy for Malaria 2016–2030 developed by the World Health Organization (WHO) [10] proposes a technical strategic framework to accelerate progress towards malaria elimination. The framework is based on three pillars and the third pillar states that it is necessary to: “*Transform malaria surveillance into a core intervention. Strengthening malaria surveillance is fundamental to programme planning and implementation and is a crucial factor for accelerating progress. All countries where malaria is endemic and those susceptible to the re-establishment of malaria should have an effective health management and information system in place for helping national malaria programmes to direct resources to the most affected populations, identify gaps in programme coverage, detect outbreaks, and assess the impact of interventions in order to guide changes in programme orientation* [10]”. The WHO’s framework also highlights that two technical aspects should be analysed along the path in order to achieve malaria-free status: the national health systems and their adaptability to support new features.

Tools for malaria surveillance improvement have been suggested for decision making, including human-friendly interfaces for intuitive guidance. For instance, Bui and Pham [11] presented the WebGIS, which is a system that was developed to detect malaria patterns based on spatial statistics in Vietnam and which supports health professionals in decision-making tasks, but does not provide any further information on the patients, such as recurrences of outcome after treatment.

Mali et al. [12] presented a visualization and monitoring system of malaria cases in the USA. The authors performed experiments with data from patients who were diagnosed in 2009 and compared them with the previous three years. They aimed to map malaria cases according to the infection site, parasite species, and the main therapeutic drug to identify malaria outbreaks that had eventually originated from imported cases from international travellers or local transmission focused on country level elimination.

Another initiative aimed to monitoring malaria cases in rural districts in Africa, applied SMS messaging from health professionals who reported weekly confirmed malaria cases. The “SMS for Life” project was carried out in Kenya and data was used to feed a web visualization system. Information was used by local health managers to improve the targeting of resources in a timely manner for diagnostic and treatment strategies [13].

Chisha et al. [14] developed a visualization system to gather and monitor real-time data on malaria tests in Zambia in order to provide timely treatment. The database was fed with information by the health units and sent to the municipality coordination where the decision-makers are able to plan better strategies. The authors claim that an increase in the number of confirmed cases of malaria was identified, which for the scenario studied is somewhat positive since more patients were correctly identified and would receive the correct treatment.

Notably, most of the malaria surveillance systems aim to estimate the number of patients with malaria episodes so that appropriate measures can control outbreaks of the disease. However, some authors argue that, in order to achieve the elimination of the disease, surveillance systems must look not only at a set of infected individuals, but also deeply at the particularities of each individual, and monitor the entire patient history, including possible recurrences [9].

Therefore, considering the guidelines provided by the current literature [3, 9, 12] and in alignment with the WHO Global Technical Strategy for Malaria 2016-2030 [10] and the WHO Malaria surveillance, monitoring & evaluation: a reference manual [15], this work presents the Malaria Trigram, an infographic visualization tool that has been developed to improve individual-level malaria surveillance analysis and provide health professionals and public health policymakers with user-friendly visual information that is focused on malaria elimination in the Brazilian Amazon.

## Methods

### Action research methodology

This research was conducted using an Action Research methodology approach [16] that was specifically the one proposed by Peter Checkland, i.e., the Soft System [17]. It is a methodology for the analysis and development of systems in which researchers and stakeholders participate jointly in the process of designing and developing them in an iterative way. The Soft System appears as an alternative to the systems engineering concept of the Hard System. The latter involves systems that, given an explicit definition of an objective, the system will be conceived (engineered) for that particular purpose and under various constraints (budget, legal, environmental). In many scenarios, this approach can be applied to troubleshooting, but real-world systems with a multi-layered process, like the Brazilian National Health System (SUS), are much more complex. Another factor for choosing the Soft System methodology is its integrated relationship with the human actors in the systems. For Checkland [16], there is a special kind of system, human activity systems, which is intrinsically linked to human action and, because of this, it cannot be reduced in its complexity to create a model. Systematic human action, as in a health system, does not behave like a natural system or an autonomous system.

The Action Research methodology requires the active participation of the researchers in the problem solving. The researchers are interested in the final result and its validation in the real world scenario with all the system’s stakeholders. The stakeholders in this case are researchers and managers that use data from the SIVEP-Malária system to make decisions and understand the malaria situation in the Amazonas state.

**Fig. 1:**
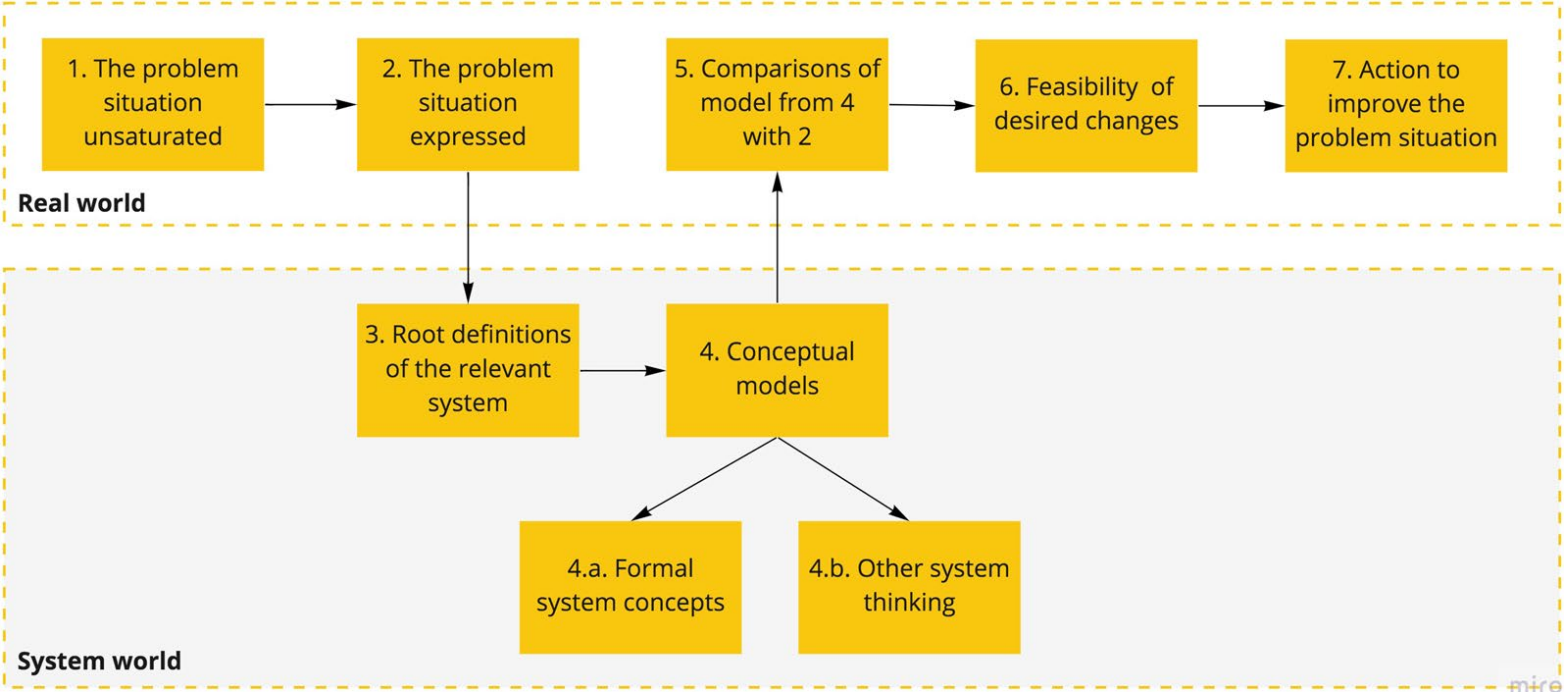
Activities and flow of the cycle. Adapted from McManus [18]

The analysis for this work is based on Checkland’s iterative learning cycles [16] (Fig. 1). The first activity is the production of a clear picture of the business process. This is a multi-perspective system analysis, with a diagrammatic system representation called “rich picture” as its output. Some root definitions are proposed, which represent a core need that should meet an actual and specific systematic complexity. After this cycle, the researchers and the stakeholders jointly build a concept model for the system. This model is validated formally and within the interaction with others related systems. Then, the model is compared with the initial state of the problem and is validated if it fits the overall system’s needs. At the end of each cycle, there is small output (new product, tool or concept) that iteratively addresses issues elucidated in the previous cycle. This new reality (new rich picture) is the input for the next cycle.

### Learning cycles

**Fig. 2:**
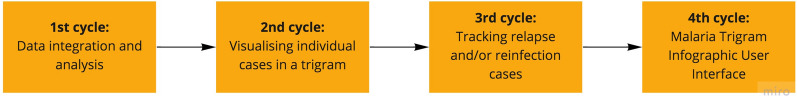
Software System iterative learning cycles

Fig. 2 presents the four learning cycles resulting from the interaction between researchers and stakeholders for elucidating and solving the target problem. Each cycle has its specificity and own contributions.

### 1st cycle—data integration and analysis

According to the WHO’s Global Technical Strategy for Malaria [10], having fast and accurate information regarding malaria epidemiological data is essential for monitoring and being successful in eradicating the disease. This information includes data on available resources (financial, personnel and infrastructure), research (in health facilities, households and pharmaceutical efficacy) and affected populations, including parasites rates and factors associated to risk groups. Another important factor about this information is related to the integration of data between the public and private sectors.

According to M’ikanatha et al. [19], an ideal system for malaria surveillance should collect and transmit data quickly, in addition to being able to receive data from other existing surveillance systems with prompt responses. Ohrt et al. [9] state that it is necessary that the system make use of minimum essential data to report a case of malaria. The paper also states that supervision of surveillance systems should be carried out by specialists in epidemiology and information technology, in order to better analyse and interpret the data.

Following guidance from the literature, one of the biggest challenges was to promote the best use of the existing data provided by SIVEP-Malária system. Any new change to the health information system is very expensive and it takes a long time to be propagated through the whole Brazilian healthcare system. Therefore, the team agreed that we should promote extracting more information from the SIVEP-Malária database without adding new primary data.

The SIVEP-Malária system was not designed for retrieving of patient follow-up data. Each entry is then predominantly classified as a new case. The Brazilian MoH guideline recommends that the patient return by the day fifth following the beginning of the treatment to verify parasite clearance, when a new microscopic exam is performed [4]. After that, any data on recurrence will only be detected through self-declared information given by the patient, and not by a query process in the information system, in the event of a new episode of illness within 60 days from a previous episode of clinical disease. Data pre-processing and aggregation on the database is required in order to identify the recurrent cases per patient and then offer some perception of the interrelated data.

**Fig. 3:**
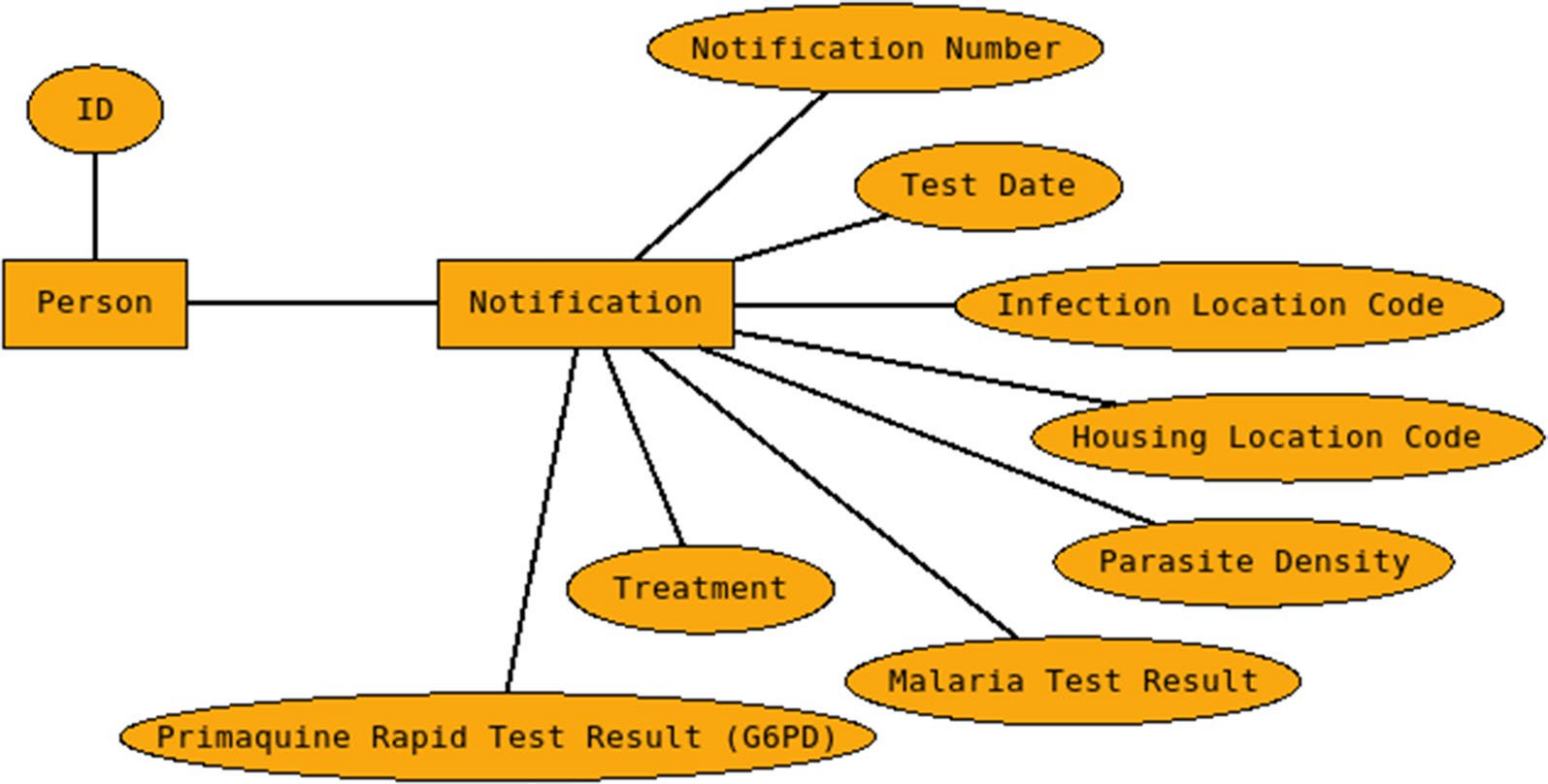
Reconstruction of the Entity Relationship Diagram from the SIVEP-Malária database, with the data used for this analysis. Source: the author, based on the data dictionary. Note that Primaquine Rapid Test Result is the G6PD test result

The data cleaning and linkage created the foundation for a strategic use of the SIVEP-Malária system. With a better understanding of the SIVEP-Malária system’s data, the first root definition of the system was defined as “*a linkage strategy tool to integrate and cache SIVEP-Malária database to analysis*”. Although the raw the SIVEP-Malária system’s database is used without any new primary data, building a cached and linked local database to produce better information was needed. This middleware was developed using Perl, with a probabilistic linkage developed in R language and the data was stored in a Sqlite3 local database. A reconstruction of the ER model of the data used is shown in Fig. 3, with the names changed to protect the database and facilitate reading and understanding.

The initial sample of the database had two cities of the Brazilian Amazon region with different characteristics and challenges [20]. One city in the state of Amazonas and the other in the state of Acre were used. The initial database had 13,660 unrelated records. These records were positive or negative for malaria and even inconclusive (null or non-existent). After the application of the probabilistic linkage, 1,514 unique identifiers were created. An analysis of the base was stratified into three major levels of similarity: from 4 to 5, from 5 to 6 and above 6. The higher this index, the greater the linkage accuracy is. The distribution of the linkage is shown in Table 1.

The probabilistic linkage technique offers a ratio (similarity rate) of how much each record appears to be from the same person. For example, if any letter is different in the name, but the other data in the register are similar, it has a higher similarity rate. If there are more dissonant elements, this rate of similarity will decrease.

**Table 1:** Distribution of the sample similarity index after probabilistic linkage

Categories	Occurrences
$4 \le N < 5$	3914
$5 \le N < 6$	3368
$N \ge 6$	6378
Total	13,660

After some data analysis, linkage ratio 6 or higher was defined as a minimal linkage quality. Then any data under this level would be use as unique data, not linked.

### 2nd cycle: visualizing individual cases in a Trigram

The second learning cycle is focused on how to create a visual way to enhance the production of information. With the new world view from the previous cycle, it is possible to understand that the visualization must represent the multivariate data from each individual patient in a time series and it must allow clear and fast comparison, be simple to understand and have a short learning curve.

Thus, the second root definition was defined as “*a visual representation of a patient’s malaria record, in a timely way in a yearly time frame with the SUS data*”. Considering the data complexity and multivariate nature, the infographic perspective was chosen as a visualization strategy [21]. Infographics have an exploratory nature through interaction, which is one of the major objectives of data visualization [22]. The double cognitive experience is one of the elements favoured by interactive infographics. The human brain reacts well to the simultaneous usage of motor (hands) and visual (graphic) elements at the same time and in the same context [23, 24]. The conscious perception part (vision-to-perception) and the call to action (vision-to-action) are activated simultaneously when using infographic interfaces. This dual mental stimulation is especially useful in systems, such as health systems, that depend on high specificity and precision from users. The ability to communicate large amounts of data and variables visually and interactively is critical for the usefulness of a health system, and causes the end user to perceive relevant events and to act as desired when facing a complex data environment [23].

### 3rd cycle: tracking relapse and/or reinfection cases

The third learning cycle is a refinement of the concepts presented in the previous ones, and is focused on the analysis of recurrent notifications of a positive test. Understanding what happens when a second or further cases of malaria happen in a year is a key information that the infographic should present.

The third root definition was defined as: “*a visual representation of a patient’s malaria record, in a timely way, that can present the dynamics of relapse and/or reinfection in a yearly time frame based on the SUS data*”. The second positive notification required new data processing and representation.

### 4th cycle: Malaria Trigram infographic user interface

The fourth cycle is the composition of a prototype of the end product. Lourenço et al. [25] conducted a standardised surveillance system landscaping in 16 countries committed to malaria elimination. The authors identified surveillance-related gaps and delineated recommendations to improve such surveillance systems. Based on the assessment, they encourage the deployment of user-friendly dashboards with indicators that are relevant to decision-making, and that are accessible and tailored to different purposes. Considering all the development in data analysis and visual representation, it was agreed to gather all of these features together in an single application. The data input has a delivery rate that varies from real time (inserted in the system at on admission of the patient to the hospitals in the capital city) to delayed buffered insertion (a bunch of paper forms that will be inserted when the healthcare practitioner returns from a riverside village to a more organised place). This makes the system’s time-to-action vary, according to the data entry, especially in the Brazilian Amazon, where the time span of the system’s data insertion may vary from real time to up to 10 days delay from notification date. So, as the first iteration of the infographic user interface, the team agreed to build an interface that could handle any flow of data insertion and help the higher level decision-makers understand the dynamics of the malaria situation accordingly to any region’s time span.

Then the following root definition was defined “*an executive information system interface for exploratory infographic data analysis with several visual representation of a patient’s malaria record, in a timely way, which can present the dynamics of relapse and/or reinfection in a yearly time frame using the SIVEP-Malária data*”. The data exploration and its outcomes might be useful for anyone of the healthcare system, but it is not feasible to do it in this cycle. The focus is an overall comprehension of the dynamics of malaria in the Brazilian Amazon region from a broad overview to a specific case-to-case analysis. This information perspective will help the policy makers understand the dynamics of each city and make decisions based on it in order to act in the best possible time span.

## Results

### Malaria Trigram

The Malaria Trigram was designed as an infographic representation of a patient’s malaria health record narrative through a time series. In this paper, the term Trigram is used as a neologism derived from a musical pentagram. This familiarity with a musical score aims to shorten the learning curve and make it more easy to read and understand. Each Trigram presents a comparable view of a patient in relation to results of malaria tests in a time series. By definition, it is a multivariate and interactive time series that relates the data of a malaria tests and its treatment in a visually simple and comparable way.

**Fig. 4:**
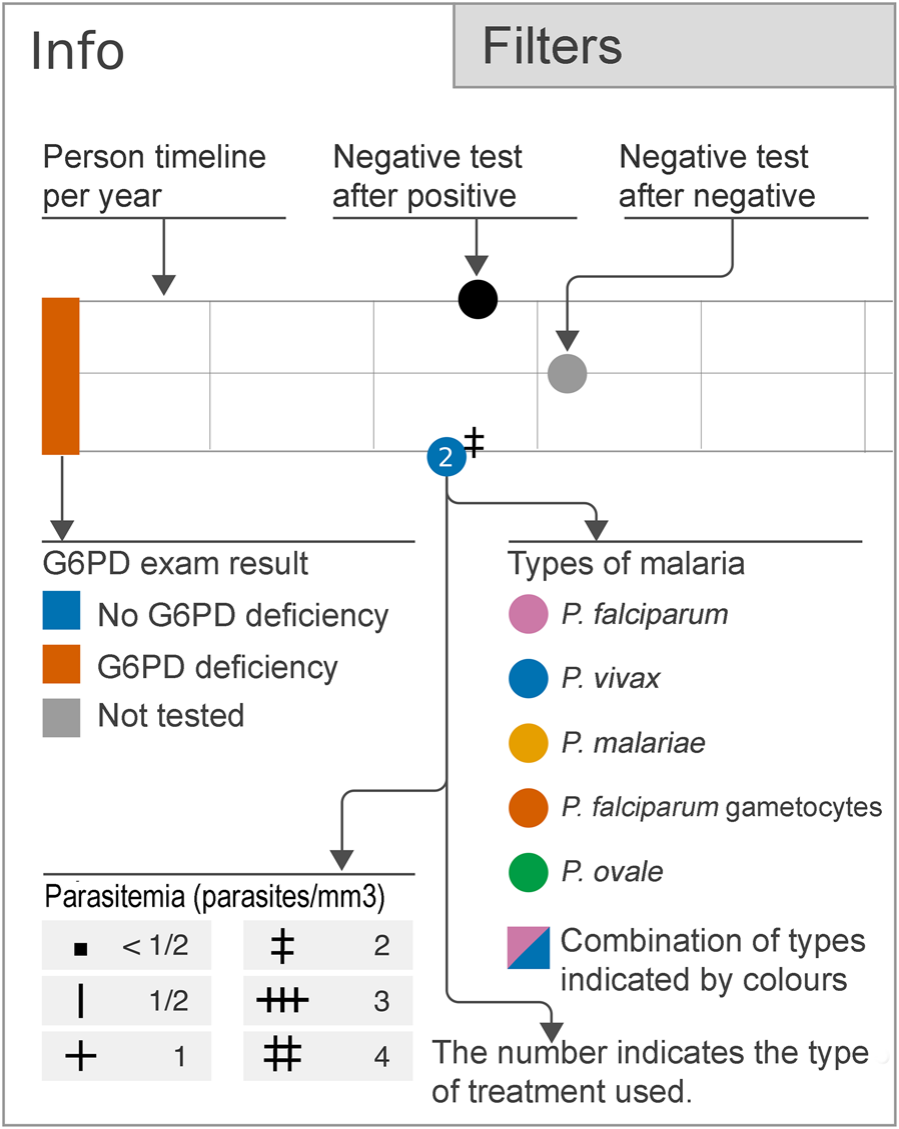
Visual structure of a Malaria Trigram

Each Trigram represents the data of a patient using icons in three parallel lines with a coloured rectangle at the beginning. The time series is divided vertically by months in a yearly span. Each element represented in the Trigram is positioned (a) horizontally in relation to the date of the malaria test result, and (b) vertically in relation to the results of the test (see Fig. 4).

The test is colour-coded to reinforce its meaning and facilitate reading. The colours chosen are readable by those who suffer from colour blindness people and tend to avoid eye fatigue. A negative test for malaria may be coloured with black (negative test after a positive one) or grey (negative test after a negative one) while positive tests for malaria may be coloured with pink (*P. falciparum*), blue (*P. vivax*), yellow (*P. malariae*), orange (*P. falciparum* gametocytes) and green (*P. ovale*), which represent different types of malaria. There is also the possibility of having a combination of malaria types with different colours, and in this case, the icon is not a circle, but a square. In most of the cases in our sample, it represents a registration/notification error.

**Fig. 5:**
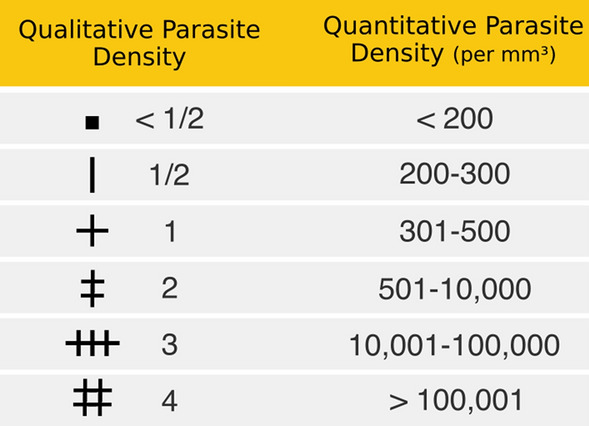
Quantitative parasite density per mm^3^ according to [26]

The number inside the circle indicates the type of the malaria treatment prescribed to the patient and the indicator at the top of the circle represents the parasitaemia (in parasites/mm^3^). Fig. 5 presents the symbols and their respective quantitative parasite density per mm^3^, according to [26].

**Fig. 6:**
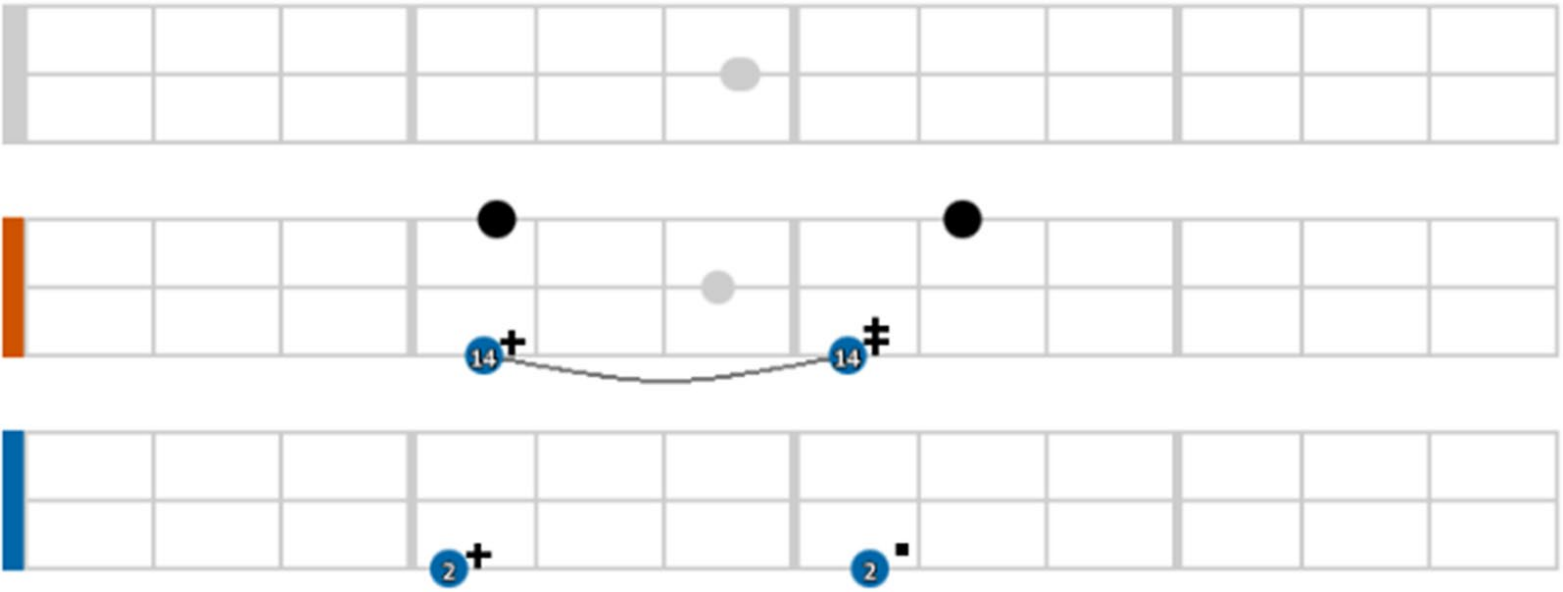
Examples of G6PD status in three different patients

The G6PD test result can be seen as the coloured rectangle at the beginning of the Trigram: blue, for normal G6PD status, orange for G6PD deficiency and grey if not tested. Fig. 6 shows an example that represent these three different cases. This visualization was pointed as critical by the stakeholders, as they can easily track the treatment and its impact on the patient’s health.

### Tracking relapses and/or cases of reinfection

Recurrences of *P. vivax* malaria consist of three categories: recrudescence, relapse, and reinfection. Recrudescence is the failure to clear asexual blood stages from the circulation, and is related to ineffective blood schizonticidal treatment. Relapse involves the re-emergence of the disease resulting from the activation of hypnozoites from the liver. Reinfection, in turn, consists of a new infection acquired by the individual through the bite of an infected vector [27].

The application of Trigram can be a useful tool for distinguishing between different types of recurrence in *P. vivax* malaria (recrudescence, relapse and reinfection), as it allows us to estimate the time elapsed between episodes of malaria in the same individual. In general, as chloroquine levels remain at therapeutic concentrations until day 28 after starting treatment, recording a new episode within this range would characterise chloroquine resistance [6]. On the other hand, after that time, recurrences can be new infections or relapses. In the Brazilian Amazon, relapses usually occur between 60 and 120 days after the index episode [28]. After that time, recurrences are likely to be mostly due to new infections. With the information that the patient has not returned to a transmission area, however, it is possible to accurately identify a relapse.

The data analysis was the foundation of this new learning cycle. It is necessary to calculate the interval between the notification after the first one and classify it according to the following tags: “less than 5 days”, “between 6 and 28 days”, “between 29 and 42 days”, “between 43 and 90 days”, and “between 91 and 180 days”. This interval classifies the second and any further cases as either a relapse or a reinfection. [6] Another important new data analysis was the geolocation of the infection. Now, the data analysis also considers if the second or any further case happens in the same city or if the patient was infected in another region and brought it to his/her location. The presence of an infected individual is capable of triggering outbreaks of malaria, thus reestablishing local transmissions where there was no more transmission. The same mechanism can occur in regions where the number of cases of malaria are being reduced. Often, in these cases, hot spots of malaria transmission are observed. For this case, the Malaria Trigram provides a visualization feature in which it is possible to geographically monitor where the patient is being infected.

**Fig. 7:**
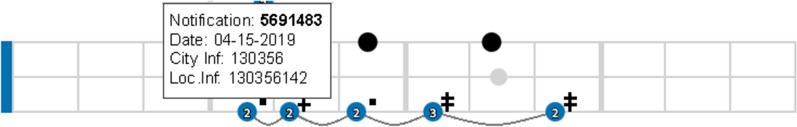
Relapse and/or reinfection case

The tracking of relapse or reinfection cases is visualised through the horizontal distribution of the circles along the time series. If there is a line arch connecting two positive tests (like a tie in a musical score), it means that the case was reported in the same city. Fig. 7 presents a patient with positive test for vivax malaria that occurred 5 times in the same city (which signifies that this city presents many opportunities for malaria infection) and also had a negative test in between the cases.

From this example, some interpretations are possible and could be investigated further: (a) the first two notifications seem to be a vivax relapse due to the short time frame and the latter notifications seem to be a reinfection; (b) the vivax malaria may have relapsed and the treatment was not efficient in this patient anymore; (c) the tests could be showing a false negative result; and (d) low adherence to treatment among other possibilities.

**Fig. 8:**
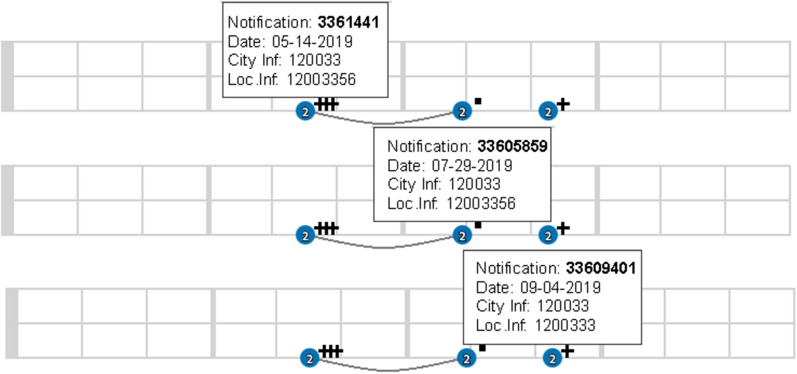
Relapse and/or reinfection case moving across cities

Fig. 8 presents a patient that presented a positive test for vivax malaria three times, but the last infection occurred in a different city from the previous ones (see the information about Loc.Inf. in the hintbox). This means that the patient was either infected in different locations or that the patient has moved. Note that, in this case, the patient did not present any reports of negative tests for malaria.

From this example, some interpretations are possible and could be investigated further: (a) after cure, the patient did not come back to the hospital for a new test and update of the medical records; (b) the patient did not take the medicine and was not cure; and (d) the patient is being reinfected; or some other possibility.

**Fig. 9:**
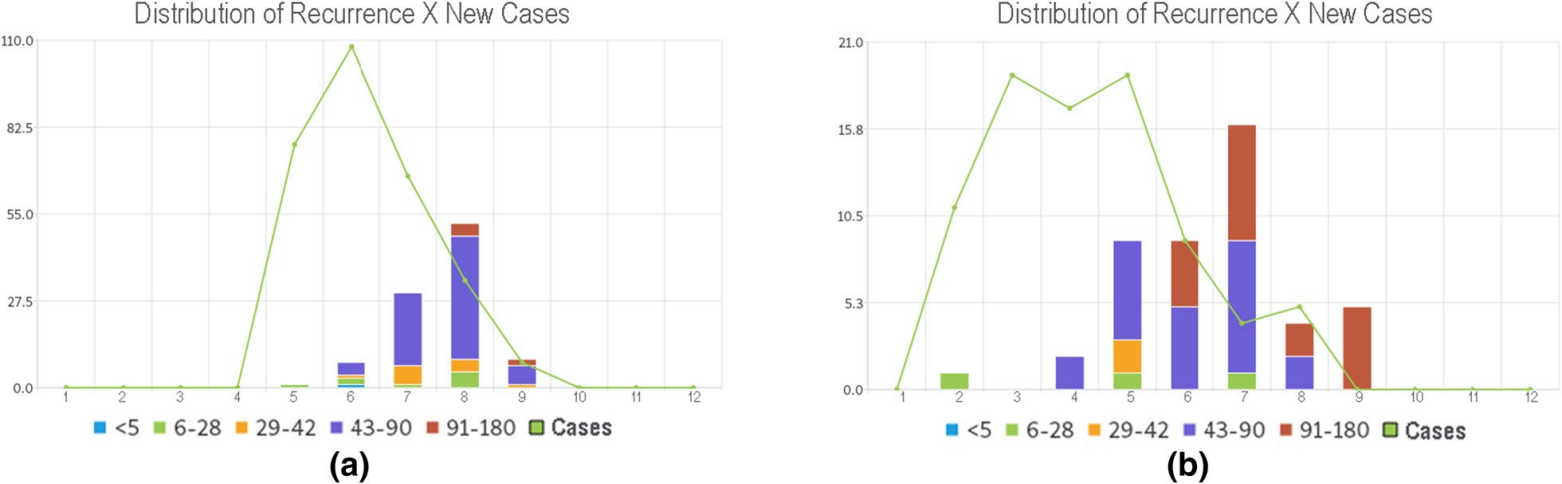
Examples of recurrence surveillance graphs for two cities

The new system proposed in the third cycle also presents a graph comparing the distribution of cases of recurrence and initial cases. Fig. 9 is an example of this graph in which the green line represents the occurrence of initial cases and the coloured columns are the number of cases of recurrence divided by the time frame of the recurrence (in days). This new graph represents the distribution of the initial cases and reinfections/relapses at the same time, thus, the user can perceive, in an aggregated way, the dynamics of relapse and reinfection in a specific city. This is a very powerful feature for analysing and comparing the dynamics of malaria distribution on a city level or in an even smaller perspective.

In Fig. 9, these two cities have a different month for the onset of the malaria outbreak. Probably, this is related to differences in the rainy seasons. The city represented in Fig. 9a has a smaller outbreak (5 months) than the city represented in Fig. 9b (8 months). City B has more reinfections over 90 days but city A has more cases in a month than B, which has its cases distributed over a range of 8 months span. The use and interpretations of such graphics might be invaluable to malaria surveillance in the Amazon region, both in real time and for historical analysis.

### Malaria Trigram infographic user interface

**Fig. 10:**
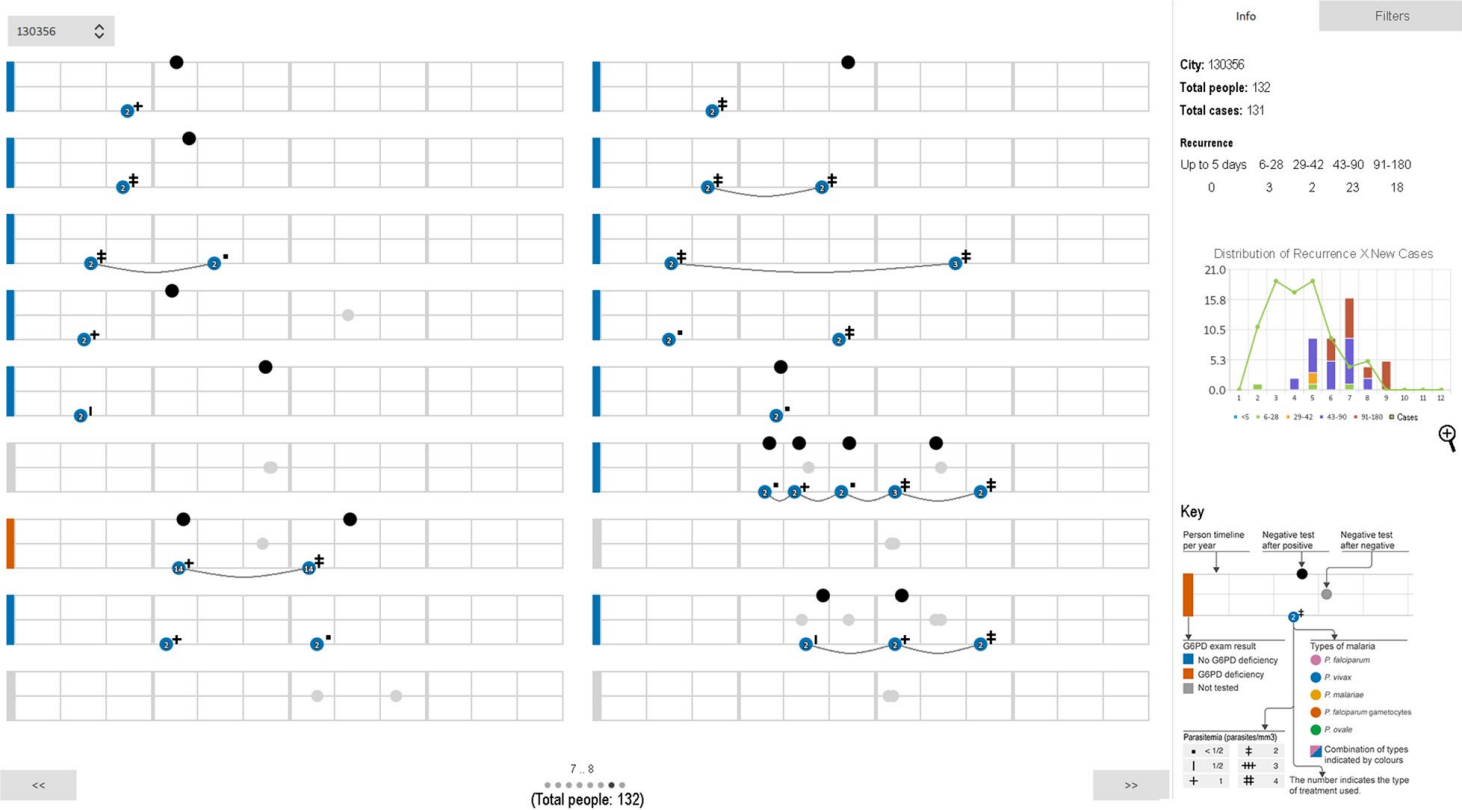
The Malaria Trigram Infographic User Interface. The interface presents individual Trigrams for each patient (on the left side, occupying the main space of the screen) and some additional information and navigation tools (on the right side). The right side of the interface gathers the quantitative reinfection/relapse analytics and an aggregate graph view of the data is presented in the Trigrams

The Malaria Trigram Infographic User Interface is a stand-alone interface (Fig. 10) that queries a local database, and is composed of linked data from the SIVEP-Malária database. The aggregate graph view has a magnifying option to better perceive the detail of the graph. This overall information and with a different representation enhances the infographic perception of the data. The user is able to perceive the big picture on the right side and explore the data on the left side of the interface.

**Fig. 11:**
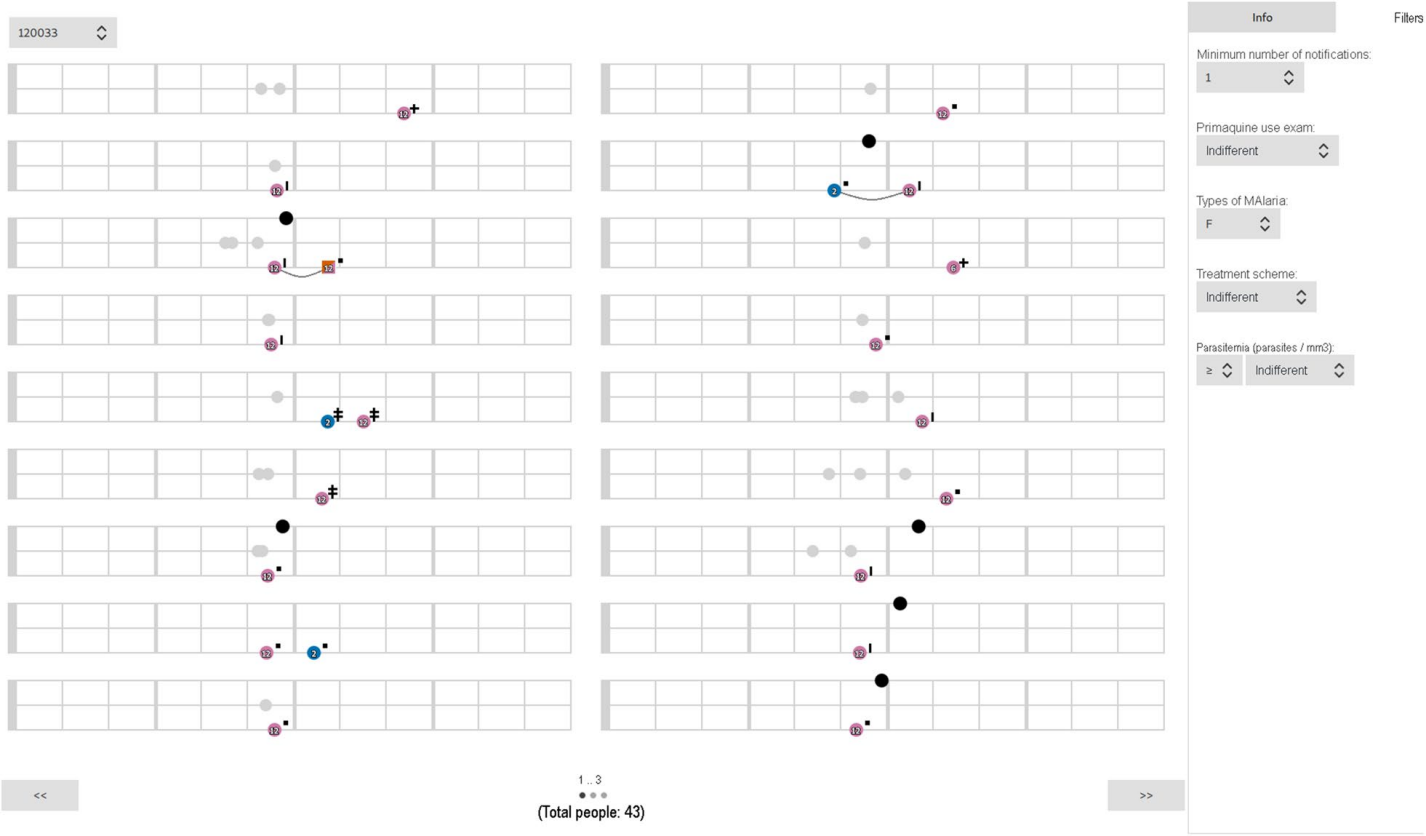
The Infographic User Interface interface with filter

To refine the visualization, a filter tab was proposed. This set of filter elements classify and reshape the visualization according to the parameters defined by the user. The filter feature allows the users to explore the visual information according to their hypothesis. In the example presented in Fig. 11, the user has selected patients diagnosed with *P. falciparum* malaria. The filter parameters are the number of records (positive or negative tests), the G6PD status, the malaria type, the treatment and the parasitaemia (parasites/mm^3^). The combination of such parameters might detect specific cases and enhance the case investigation.

The last visual element of the infographic interface is a key to the Trigrams on the lower lower corner. The Trigrams are built to be simple to read, but we understand that this takes time to get used to it, so this key assists in the shortening the learning curve. This visual aid is a strategy to train the user to visually enhance the perception of the dynamics of the Trigram.

At the end of this cycle, a functional prototype was presented to all stakeholders and the overall feedback was positive, with one suggestion in regards to the parasitemia; that it is a clinical data that’s not very significant in the overall analysis. This input can cut out some visual saturation and will be considered in the next cycles.

## Discussion

The analysis of data is the basic change promoted by our work. By using the same SIVEP-Malária system to create new and more useful information than just generating plain reports, our analyses were well received by the stakeholders. The participatory learning process enhanced the usefulness of the data, since relevant data was identified with the assistance of the stakeholders. The parameters and knowledge are based on the current theory and policies of the surveillance team. In January 2020, a new Brazilian protocol for malaria treatment was launched. The periodic review of this treatment protocol is important in order to update the most appropriate and safest drugs for the treatment of each species of malaria [26]. Our tool, the Malaria Trigram, in conjunction with the current Brazilian surveillance systems can also be used during this review process as an auxiliary tool for following-up and analysing the results of such interventions and drug sensitivity testing. As a result, as new theories, policies, and perspective to surveillance change over time, new elements can be added to the analysis.

The Malaria Trigram should have a great impact on public health policies in the state of Amazonas, as it presents qualified information on pre-existing health data, without the need for a new base or any change in the current malaria surveillance system. As presented, based on historical patients’ data, the Malaria Trigram allows for the identification and analysis of *sui generis* cases per patient or per region, and gives the opportunity of triggering timely control interventions in order to reduce or eliminate transmission, and, as such, it is a cost-effective alternative.

The infographic presentation of the data allows the user to vertically compare the distribution behaviour of malaria infections. Using the filter tool to select specific subgroups, this comparison can be stratified according to the user’s on-demand needs. The perception of outliers, both in the case reinfection frequency and date, can be represented through visualization for further investigation. Thus, the visualization should enhance the healthcare authority’s active search for specific cases and patients.

Another relevant contribution of this work is that a visual representation of the total distribution of the cases during a year. The reinfection/relapse graphic is an invaluable information for benchmarking the cases and their distribution during several yearly series. Distribution can also be compared among or between cities, considering geolocation, weather, and population, and new metrics can emerge for such analysis, such as the days difference between the normal peak of initial cases and the normal peak of the reinfection. This information should assist new public health policies and healthcare strategies over time.

The willingness and openness to change of the stakeholders have helped this research to achieve good results in a short time. The participatory methodology was well suited to the complexity of the research and the extremely specific domain of the malaria surveillance required a close relationship with the stakeholders. The final outcome is a specific solution to malaria in the context of the Brazilian SUS and contemplates the complexity of Amazon region. We believe that some of that outcomes can be adjusted to the new context, with some cycles of refinement. However, this visualization is not generically conceived to fit any needs. The relevance to the final user is a validation that is more relevant than an unspecific general visualization. Thus, there is an opportunity to develop domain specific infographics for healthcare in a narrow context, with simultaneous multiple variables, with a great impact on the production of information.

## Limitations

One limitation of this work is that the information was generated by the Brazilian SIVEP-Malária system and many problems can arise because it is not possible to ensure the quality of the data. Data quality is part of the complexity of the Brazilian health service, but it has impact on the quality and accuracy of the analyses. It is relevant to recognize and present the missing data since it can reveal important surveillance events (such as lack of test notification) and then trigger or plan monitoring actions. The linkage process is also very noisy, as the healthcare system in the Brazilian Amazon region is a big challenge in many aspects (distance, feasibility of travel, lack of resources to reach the more isolated regions, including distance). Even a simple record as name and mother’s name is noisy, because the way that the person notifies a name can change every time the person seeks assistance at a healthcare facility. To mitigate the linkage issues, we used a high score-match.

A further limitation of the environment and a difficulty of this work was the COVID-19 pandemic. In addition to all the personal issues that the pandemic caused for everyone, it made the final evaluation more difficult, because the stakeholders turned their attention to a new priority area, especially at the beginning. Thus, there could be more cycles and evaluations if the stakeholders had had a greater interaction.

## Conclusions

In this paper, the Malaria Trigram was presented; it is an infographic visualization tool aimed to help health professionals and policy makers to recognise and analyse different types of patterns in malaria events, such as relapses or reinfection, as well as with case tracking. No additional primary data collection nor changes in the current healthcare process are required.

The Malaria Trigram enables health professionals and policy makers to plan and coordinate interventions for malaria elimination that are integrated with other parallel actions, such as vector control management, developing of effective drugs for treatment and the development of an effective vaccine.

## Data Availability

The data that support the findings of this study are available from FMT-HVD but restrictions apply to the availability of these data, which were used under license for the current study, and so are not publicly available. Data are however available from the authors upon reasonable request and with permission of Vanderson Sampaio.

## References

[1] Cibulskis RE, Alonso P, Aponte J, Aregawi M, Barrette A, Bergeron L (2016). Malaria: global progress 2000–2015 and future challenges. Infect Dis Poverty..

[2] Ferreira MU, Castro MC (2016). Challenges for malaria elimination in Brazil. Malar J..

[3] Recht J, Siqueira AM, Monteiro WM, Herrera SM, Herrera S, Lacerda MV (2017). Malaria in Brazil, Colombia, Peru and Venezuela: current challenges in malaria control and elimination. Malar J..

[4] Ministério da Saúde. Guia de tratamento da malária no Brasil. Ministério da Saúde. Secretaria de Vigilância em Saúde. Departamento de Imunização e Doenças Transmissíveis. 1st ed. Brazil | Brasília - DF: Editora MS; 2020.

[5] Lana R, Nekkab N, Siqueira AM, Peterka C, Marchesini P, Lacerda M (2021). The top 1%: quantifying the unequal distribution of malaria in Brazil. Malar J..

[6] Balieiro AA, Siqueira AM, Melo GC, Monteiro WM, Sampaio VS, Mueller I (2021). Short-time recurrences of Plasmodium vivax malaria as a public health proxy for chloroquine-resistance surveillance: a spatio-temporal study in the Brazilian Amazon. Int J Environ Research Public Health..

[7] Terrazas WCM, de Souza Sampaio V, de Castro DB, Pinto RC, de Albuquerque BC, Sadahiro M (2015). Deforestation, drainage network, indigenous status, and geographical differences of malaria in the State of Amazonas. Malar J..

[8] Sampaio VS, Siqueira AM, Alecrim MdGC, Mourão MPG, Marchesini PB, Albuquerque BC (2015). Malaria in the State of Amazonas: a typical Brazilian tropical disease influenced by waves of economic development. Rev Soc Brasil Med Trop..

[9] Ohrt C, Roberts KW, Sturrock HJ, Wegbreit J, Lee BY, Gosling RD (2015). Information systems to support surveillance for malaria elimination. Am J Trop Med Hyg..

[10] WHO. Global technical strategy for malaria 2016-2030. Geneva: World Health Organization; 2015.

[11] Bui TQ, Pham HM (2016). Web-based GIS for spatial pattern detection: application to malaria incidence in Vietnam. SpringerPlus..

[12] Mali S, Tan KR, Arguin PM. Malaria surveillance–United States, 2009. 2011;. 21508921

[13] Githinji S, Kigen S, Memusi D, Nyandigisi A, Wamari A, Muturi A (2014). Using mobile phone text messaging for malaria surveillance in rural Kenya. Malar J..

[14] Chisha Z, Larsen DA, Burns M, Miller JM, Chirwa J, Mbwili C (2015). Enhanced surveillance and data feedback loop associated with improved malaria data in Lusaka. Zambia. Malar J..

[15] WHO. Malaria surveillance, monitoring & evaluation: a reference manual. Geneva: World Health Organization; 2018.

[16] Checkland P (2000). Systems thinking, systems practice: includes a 30-year retrospective. J Operation Res Soc..

[17] Checkland P, Poulter J, Reynolds M, Holwell S (2010). Soft systems methodology. Systems approaches to managing change - a practical guide.

[18] McManus JJ, Wood-Harper A (2003). Information systems project management: Methods, tools and techniques.

[19] M’ikanatha NM, Lynfield R, Julian KG, Van Beneden CA, de Valk H. Infectious disease surveillance: a cornerstone for prevention and control. In: Infectious Disease Surveillance. 2007;Chapt 1;1-17;.

[20] Brito-Sousa JD, Murta F, Vitor-Silva S, Sampaio VS, Mendes MO, Brito MA (2021). Real-life implementation of a G6PD deficiency screening qualitative test into routine vivax malaria diagnostic units in the Brazilian Amazon (SAFEPRIM study). PLoS Negl Trop Dis..

[21] Cairo A (2012). The Functional Art: An introduction to information graphics and visualization.

[22] Kehrer J, Hauser H (2012). Visualization and visual analysis of multifaceted scientific data: a survey. IEEE Trans Vis Comput Graph..

[23] Ware C (2019). Information visualization: perception for design.

[24] Jacob P, Jeannerod M (2003). Ways of Seeing: The Scope and Limits of Visual Cognition.

[25] Lourenço C, Tatem AJ, Atkinson PM, Cohen JM, Pindolia D, Bhavnani D (2019). Strengthening surveillance systems for malaria elimination: a global landscaping of system performance, 2015–2017. Malar J..

[26] Ministério da Saúde. Boletim Epidemiológico Malária. Secretaria de Vigilância em Saúde; 2020. Available from: https://www.gov.br/saude/pt-br/media/pdf/2020/dezembro/03/boletim_especial_malaria_1dez20_final.pdf.

[27] Mueller I, Galinski MR, Baird JK, Carlton JM, Kochar DK, Alonso PL (2009). Key gaps in the knowledge of Plasmodium vivax, a neglected human malaria parasite. Lancet Infect Dis..

[28] Vitor-Silva S, Siqueira AM, de Souza Sampaio V, Guinovart C, Reyes-Lecca RC, de Melo GC (2016). Declining malaria transmission in rural Amazon: changing epidemiology and challenges to achieve elimination. Malar J..

